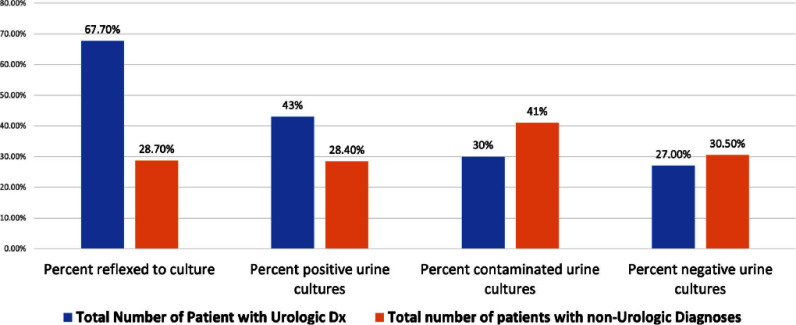# The “Streamlining” Initiative - Reduction of Unnecessary Urine Testing in Patients Presenting at CRH

**DOI:** 10.1017/ash.2025.242

**Published:** 2025-09-24

**Authors:** Ioana Chirca, Whitney Brown, Rebecca Worley, David Cox, Genie Hamilton, April Dukes, Diana Wilkerson, Michelle Thompson, Amanda Smith, Sheila Fussell, Baily Edmondson, Kim Pearson, Hope French

**Affiliations:** 1 AdventHealth; 2 Crisp Regional Hospital; 3 Crisp Regional Hospital

## Abstract

**Background:** Crisp Regional Hospital (CRH) in Georgia initiated a quality improvement project to address the excessive and inappropriate urine culture orders among inpatients. The project aimed to reduce these orders by at least 30% by June 2024, targeting the prevalent issues of increased healthcare costs and antibiotic misuse stemming from unnecessary testing. **Methods:** Using the Institute for Healthcare Improvement (IHI) framework, the project implemented a series of multidisciplinary strategies. These included nursing education on proper urine collection, policy updates to facilitate accurate specimen collection from specific patient groups, medical staff education on appropriateness of urine test ordering and a change in reflex criteria for urine testing. Data was analyzed using statistical process control and T-tests to assess the impact of the interventions. **Results:** The intervention led to a reduction in urine tests from 618 to 570. Tests reflexed to culture decreased significantly from 34.63% to 18.95% (p **Conclusion:** The quality improvement initiative at CRH significantly reduced unnecessary urine cultures, optimized resource use, and maintained diagnostic integrity. The interventions implemented were effective and scalable, demonstrating substantial cost savings and enhanced patient care quality. Further efforts will focus on analyzing the impact of removing pre-checked orders and implementing mandatory testing indications to continue improving urine testing practices.

**Figure:**
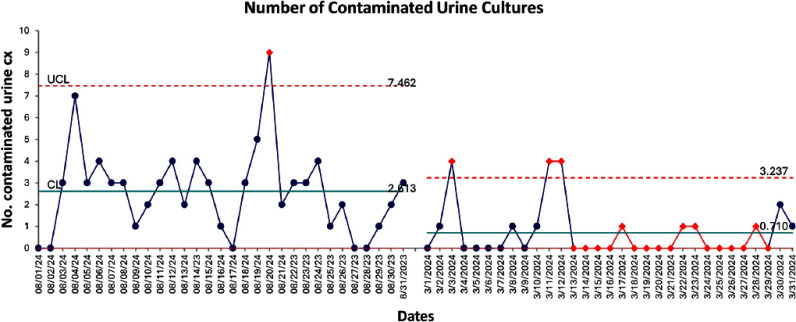

**Figure:**
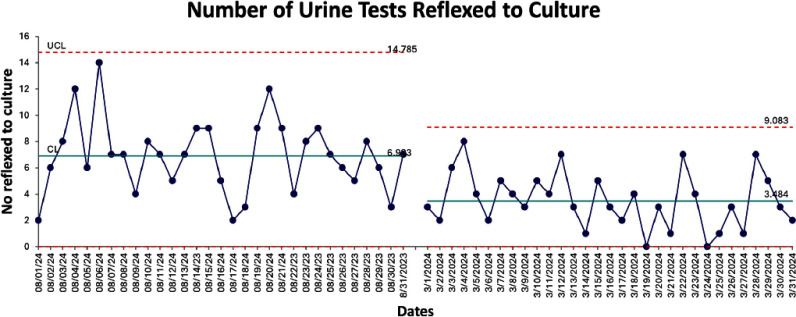

**Figure:**
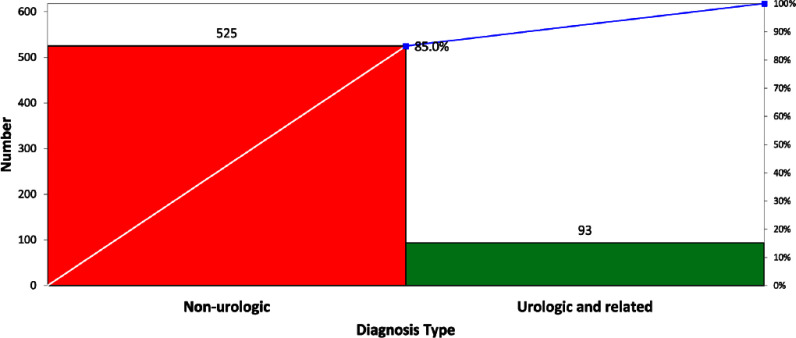

**Figure:**